# Process-based approach to modeling recurrent-event data explicated on the basis of occurrences of tooth losses in two different prosthetic treatment concepts

**DOI:** 10.1186/s13063-016-1360-y

**Published:** 2016-05-17

**Authors:** Hans H. Diebner, Birgit Marré, Ingo Roeder, Michael H. Walter

**Affiliations:** Faculty of Medicine Carl Gustav Carus, Technische Universität Dresden, Institute for Medical Informatics and Biometry, Fetscherstrasse 74, Dresden, D–01307 Germany; Department of Prosthetic Dentistry, Faculty of Medicine Carl Gustav Carus, Technische Universität Dresden, Dental School, Fetscherstrasse 74, Dresden, D–01307 Germany

**Keywords:** Recurrent events, Cumulative sample history function, Shortened dental arch, Dental prosthesis

## Abstract

**Background:**

In studies comparing different prosthetic treatment concepts the repeated loss of teeth was chosen as the primary outcome. The resulting data appear to represent a data structure of recurrent events. However, the application of an existing method for recurrent events is far from straightforward. Often only the first event or the final state is analyzed using Kaplan–Meier survival statistics, thereby giving a great deal of information away.

**Methods:**

The paper presents a strategy for the analysis of recurrent data using a previously published study on the influence of different prosthetic treatment concepts for the shortened dental arch on tooth loss. A method based on cumulative sample history functions of recurrent events was adjusted for tooth loss. The shapes of these cumulative functions suggest a time dependency of the recurrence rate. To keep the model as simple as possible, a tripartite Poisson process (which assumes piecewise time-independent rates) was fitted to the cumulative mean functions stratified by treatment.

**Results:**

Within the middle interval of the three-phasic process, the treatment effects differ significantly, which is interpreted as a delay of tooth loss due to the use of one type of prosthesis (fixed) compared with the other (removable).

**Conclusions:**

An analysis based on cumulative history functions is based on process, therefore, temporally changing characteristics are better captured than in methods for survival analyses. The presented approach offers useful new insight into the temporal behavior of ongoing tooth loss after prosthetic treatment.

**Trial registration:**

The trial has been registered at controlled-trials.com under ISRCTN97265367 (registration date 4 April 2008).

**Electronic supplementary material:**

The online version of this article (doi:10.1186/s13063-016-1360-y) contains supplementary material, which is available to authorized users.

## Background

### Repeated loss of teeth as recurrent events

The paper presents an adapted approach to recurrent data analyses based on a previously published study on the influence of different prosthetic treatment concepts for the shortened dental arch. The primary outcome measure was tooth loss [1–3]. Over time within the study population repeated tooth loss occurred. This repeated loss of teeth appears to represent a data structure that matches approaches for analysis of recurrent events. However, there are intricacies that prevent easy implementation of existing recurrent-event time models.

For example, in observations of multiple times to tumor formation in the context of carcinogenesis experiments (see the seminal paper by Gail et al. [4]), a legitimate question is whether there has been a relapse of a removed tumor or a further tumor at another location. Likewise, it is arguable whether a subsequent loss of a tooth can be interpreted as a recurrent event. Of course, a class of events for successive losses of teeth can be constructed, yet there are striking differences compared to potentially infinitely many recurrent tumors. Firstly, the number of teeth is finite. Secondly, unlike an assumed complete recovery from a tumor, the previous state cannot be restored after tooth loss.

A related question is how left-censored data should be treated. Patients enter the study with a given number of teeth. The event times of precursive losses are, in general, unknown, but the number of precursive events should mostly be clear when taking the maximum number of teeth into account. Thus, methodological adaptations with respect to previously published methods are necessary.

Tooth loss as described within this study is actually the result of medically indicated extractions. Often more than one tooth at a time has to be extracted. Many methods for recurrent events assume non-simultaneous events, particularly, if the analysis is based on gap times. Although the extractions were well founded, the extraction times are in a sense artificial compared to natural loss. Both natural and interventional losses may worsen the conditions for the residual set of teeth.

### Hazard rate vs recurrence rate

The analysis of multiple times to event data is a rather young statistical field of study and still immature. WB Nelson [5] offers an intelligible nonparametric access and elaborates on the yet short history of recurrent-event data analysis. RJ Cook and JF Lawless [6], in contrast, focus more on parametric approaches.

A majority of recurrent-events models are based on extensions of Cox’s survival model, focusing on hazard rates [6, 7]. Thereby, models that are based on total time to event as well as on gap time have been used. The former models assume incidences in a continuous manner whereas the latter models assume a reset after each event into a ground state paired with a reset of the stopwatch for the next event. The gap time approach appears to be improper for a description of the shortened dental arch data since there is definitely no treatment leading to complete recovery of the patient that allows a reset of the waiting time for subsequent losses of teeth. Even more contraindicative for the use of gap times is that, quite often, several teeth have to be simultaneously extracted for medical reasons. That would lead to zero gap times. Therefore, survival models based on gap times cannot be applied.

The continuous total time to event approach may also be misguided, since a loss of teeth may worsen the condition for dental conservation with respect to the residual set of teeth. Either an accelerated failure time model and/or a model that accounts for the numbers of extracted, as well as residual teeth, seems to be more appropriate. In any case, there is no straightforward application of a proper survival model with respect to the data at hand.

As Nelson emphatically points out, recurrence rates of systems should be carefully distinguished from hazard rates for the lives of non-recurrent items [5]. However, the distinction loses its stringency for renewal processes, where a failure of an item can be set back (repaired) to the item’s original state. That is not the case for tooth losses. From the perspective of tooth populations, a loss represents a death. From the patient’s perspective, a tooth loss has the characteristic of a recurrence. However described, recurrent events may be addressed within mixed survival models (frailty analysis) or by clustering the recurrent events of a unit (e.g., a patient). In contrast, the following analysis is based on existing nonparametric methods [5]. Attempts to perform frailty analyses produced unconvincing results, which are not shown here. Features of the available data that argue against mixed survival models are addressed in subsequent sections. Most of the aforementioned problems typically related to hazard-based approaches can be circumvented when a process-oriented stance based on an instantaneous event intensity function is taken. A piecewise constant Poisson process is modeled, rendering the method parametric. All calculations and presentations have been performed using R [8].

## Methods

### Study design

The data set originates from a multicenter randomized controlled clinical trial with two parallel groups and an allocation ratio of 1. The intention-to-treat principle was applied. The trial design has been published in detail [1]. The study jaw (mandible or maxilla) was determined by the dentition present. All molars had to be missing with at least the canine and one premolar present on each side (a shortened dental arch). In one group, the patients were provided with a partial removable dental prosthesis for replacement of the missing posterior teeth (treatment 1 in the following). In the other group, they were treated according to a concept that basically aimed at preserving the shortened dental arch without complete tooth replacement using a fixed prosthesis (treatment 2 in the following). The primary outcome was tooth loss. All losses were recorded for the whole dentition, for both the study jaw (SJ) in which the study treatment was delivered and the other jaw, the non-study jaw (NSJ). The required sample size was calculated a priori using a log-rank test based on an expected tooth loss rate of 20 % for treatment 1 and 5 % for treatment 2 after 5 years applying a two-sided primary significance test (*α*=5 %). Loss to follow-up of patients over time was assumed to follow an exponential distribution, the dropout rate adding up to 10 % of recruited patients after 5 years. The maximum type I error of the applied two-sided significance test was set to 5 %. The power was set to 75 % in terms of detecting treatment differences of the above magnitude. If the differences were not pronounced, the power of 75 % was expected to be sufficient to at least demonstrate the equivalence of the shortened dental arch. According to the presumptions and requirements, the calculated required number was 70 patients per treatment group. After the occurrence of target events, the follow-up examinations were continued and further tooth losses recorded. For the primary outcome, the 3-year and 5-year results have been published based on Kaplan–Meier survival analyses [2, 3]. Although the 5-year analysis was planned to be final, the study team agreed to extend the observation period to 10 years. During the prolongation of the observation period, further tooth losses occurred, i.e., recurrent events. These appear to be significant for describing the long-term outcome of the treatments. The current paper is based on the 8-year data. Of the 215 randomized patients, 90 patients attended the 8-year examination (Additional file 1). The trial has been approved by a research ethics board (TU Dresden, EK 260399). The informed consent of the participants was obtained.

### Data description

**Fig. 1:**
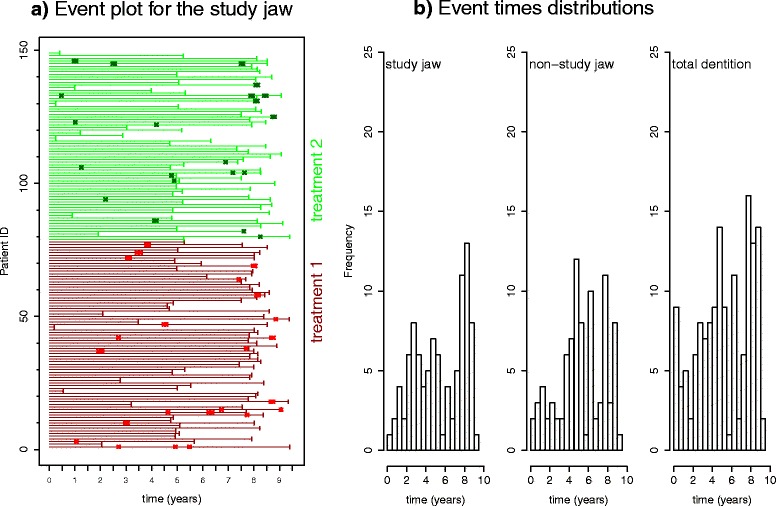
**a** Event plot for teeth extraction events in the study jaw. Each *horizontal line* represents the duration of observation of a patient. It ends either with the eventual tooth extraction or with a right-censoring time given by a final examination. The start time (inclusion into the survey) of each patient has been set to zero. IDs 1 through 78 are patients treated with a partial removable prosthesis (*red lines with cross markers* at the extraction times). IDs 79 through 149 are patients treated with a fixed prosthesis (*green lines and markers*). Multiple events occurring on the same day are slightly shifted for better visibility. **b** Histograms for the event times (only primary outcome, i.e., tooth loss) calculated separately for the study jaw (*left*) and the non-study jaw (*middle*) as well as for the pooled data (*right*)

To get a surface impression of the recorded tooth extraction data, we follow the recommendation by Cook and Lawless [6] to present an event plot. Figure 1a displays the observed events (time resolution is one day) for the two treatment groups in an event plot, whereby only the events observed in the SJ are shown. The calendrical event times with different start times have been transformed to observational periods with a common zero start time displayed on the abscissa. The ID (consecutive numbering) of the patients is displayed on the ordinate. The lower 78 IDs belong to patients under treatment 1 (partial removable prosthesis) and IDs 79 through 149 (71 patients) belong to patients with fixed prostheses (treatment 2)^1^.

The onset of events seems to be more moderate for treatment group 1 compared to treatment group 2. However, there are denser occurrences in the intermediate time interval. Eventful and uneventful intervals alternate. A histogram allows for a quick tentative visual assessment. The obvious concentrations of primary events (tooth loss) at roughly around half the total observation time and at the end is confirmed by the event time distributions shown in Fig. 1b. The empirical distributions of event times exhibit bimodal shapes, whereby the second modes are rather pronounced (temporally condensed). There seems to be an enhanced frequency of extractions about 4 years after the onset of the study and an even more temporally condensed second wave of extractions at about 8 years. Note that a large fraction of observations is censored roughly after the first half of the study (as can be clearly seen in Fig. 1a), which gives the second wave additional emphasis once more. The high frequency of censoring in the middle is because the corresponding patients declined to participate further after 5 years [1–3].

### Modeling cumulative sample history functions

**Fig. 2:**
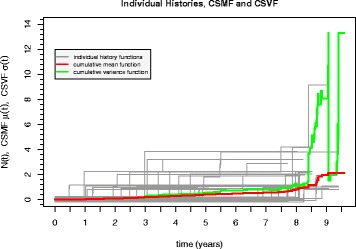
Individual and sample cumulative history functions for the SJ data set containing both treatment groups. *Gray lines*: Individual cumulative event counts (histories) with *N*_*i*_(*t*) for *i*=1,…,149 (patient ID). All curves *N*_*i*_(*t*) have a small vertical offset with respect to *N*_*i*−1_(*t*) so that overlays, particularly at zero, become visible. *Red curve*: CSMF according to Eq. 3. *Green curve*: CSVF according to Eq. 4. *CSMF* cumulative sample mean function, *CSVF* cumulative sample variance function, *SJ* study jaw

Let *N*_*i*_(*t*) represent the number of events over the time interval [0,*t*] for the *i*th out of *m* patients. The set of event histories *N*_*i*_(*t*) for the SJ of each of the *m*=149 patients are plotted in Fig. 2 together with the cumulative sample mean function (CSMF) as well as the cumulative sample variance function (CSVF) to be defined in the following. Figure 2 supports an intuition of how the individual event histories add up to shape the CSMF. The individual histories each end at their censoring time. For non-censored individual histories (subscript nc), the CSMF is defined as

1 $$ \hat{\mu}_{\text{nc}}(t) = \frac{1}{m}\sum_{i=1}^{m}{N_{i}(t)}.   $$

Similarly, the CSVF is defined as

2 $$ \hat{\sigma}_{\text{nc}}\{ N(t) \} = \frac{1}{m-1}\sum_{i=1}^{m}{\left\{ N_{i}(t) - \hat{\mu}_{\text{nc}}(t)\right\}^{2} }.   $$

**Fig. 3:**
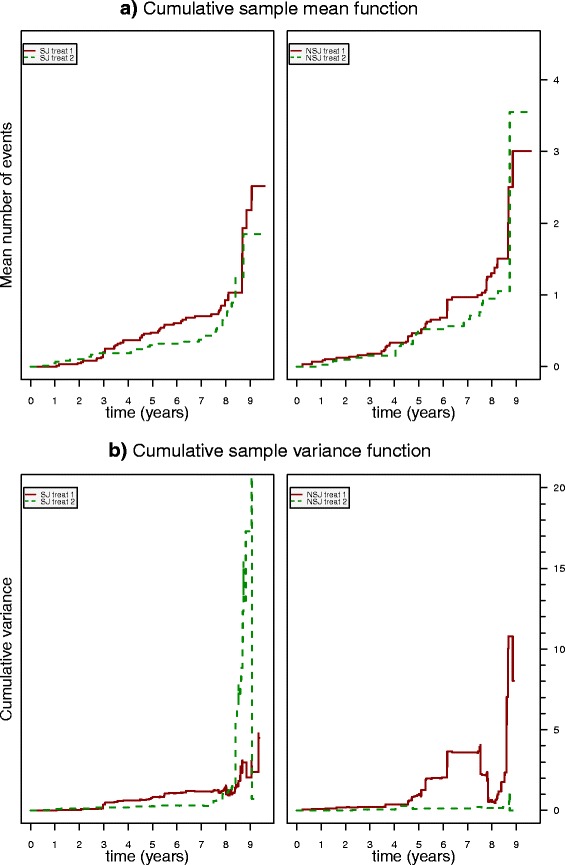
**a** Cumulative sample mean functions (Eq. 3) for the teeth extraction events stratified for treatment. Left panel: Data from SJ. Right panel: Data from NSJ. **b** Cumulative sample variance functions (Eq. 4) for the teeth extraction events stratified for treatment. Left panel: Data from SJ. Right panel: Data from NSJ. *NSJ* non-study jaw, *SJ* study jaw, *treat* treatment

However, we have to account for the different censoring times, which makes the calculation a bit more complicated. The denominator to calculate the instantaneous means decreases in time due to censoring. In this case, the CSMF can be calculated iteratively as

3 $$ \hat{\mu}(t) = \hat{\mu}(t-\Delta t) + \frac{1}{r(t)}\sum_{i=1}^{r(t)}{\left(N_{i}(t) - N_{i}(t-\Delta t) \right)},   $$

with *r*(*t*) denoting the number of patients at risk (non-censored) at time *t*. From Eq. 3 and from the corresponding graphs in Figs. 2 and 3a it can be seen that the CSMF is a monotonically increasing staircase function. Generally, the choice of *Δt* depends on the temporal resolution of event data and should be sufficiently small to avoid skipping events that are separated less than *Δt*. We choose *Δt* to be the temporal resolution of the observations, i.e., 1 day. Including censoring times, the estimated variance for asynchronously censored event data is given by

4 $$ \hat{\sigma}\{ N(t) \} = \frac{1}{r(t)-1}\sum_{i=1}^{r(t)}{\left\{ N_{i}(t) - \hat{\mu}(t)\right\}^{2} }.   $$

In contrast to the CSMF, the CSVF is not necessarily monotonous (see Figs. 2 and 3b).

Figure 3 depicts the CSMFs and CSVFs of the tooth extraction events stratified for SJ/NSJ and treatment (type of prosthesis). These cumulative functions belong to the class of cumulative history functions [5]. Nelson’s book [5] focuses on nonparametric methods based on CSMF and CSVF and serves here as the main reference for nonparametric approaches.

A preliminary rule to determine the censoring time for a given patient is to use the final examination date. However, if a patient lost his/her last tooth before the final examination, a censoring time equal to the last extraction time is introduced since this patient is no longer at risk with respect to the target variable. This does not occur with the SJ. However, for the NSJ, a couple of patients lost their last tooth before the final examination took place. Some patients (26) even entered the study with no teeth at all in the NSJ, leaving 123 patients that contribute to the analysis of the NSJ. In such cases, the choice of the SJ was predefined instead of random, thus ruling out the NSJ as a control group in a rigorous sense (besides other restrictions as, e.g., the dependency of the condition of one jaw on the condition of the other). Eventually, two distinct censoring times have to be used separately for the SJ and the NSJ.

The CSMF, $\hat {\mu }(t)$, is an estimate of the population cumulative mean function, *μ*(*t*), and quantifies the mean of the distribution of the number of events at any time *t*, as shown in Fig. 3a for the stratified records. The time course of the diversely stratified CSMFs once more confirms that there is a considerable increase in the number of tooth extractions towards the end of the observation period. At the same time, the variance increases considerably, since the number of simultaneously extracted teeth for some patients is rather high and the number of patients at risk strongly decreases.

More important than *μ*(*t*) is the time derivative *λ*(*t*)=d*μ*(*t*)/d*t*, which quantifies the instantaneous recurrence rate or intensity function. Given that the presuppositions discussed in section “Hazard rate vs recurrence rate” (continuous incidences or renewal) for an extension of Cox survival modeling are satisfied, the instantaneous recurrence rate *λ*(*t*) resembles the hazard rate in univariate survival processes, but there are also important differences. The cumulative mean function sums up the past events, whereas the cumulative hazard takes the (known) number of individuals at risk at a given instant of time into account. Both the instantaneous recurrence rate and the hazard rate are events per time unit per population unit. If one assumes a given recurrent-event process with a potentially open number of recurrences per individual, then the instantaneous recurrence rate refers to the (mean) number of individual event recurrences. For the special case of non-recurrent events, the analysis can be simplified to a standard survival analysis and *λ*(*t*) is replaced by the hazard rate for non-recurrent events. The instantaneous recurrence rate is addressed in more detail below.

The cumulative functions allow for a heuristic assessment. Regarding the SJ (left panel of Fig. 3a), for the first roughly 1100 days (≃3 years), treatment 1 (partial prosthesis) seems to be slightly superior compared to treatment 2 (fixed prosthesis). With respect to the NSJ (right panel of Fig. 3a), both treatments lead to approximately the same CSMF within the first 1100 days. The aforementioned slight advantage is not significant, though, as shown later.

In contrast to the initial 3-year interval, from *t*=1100 days onward the two curves stratified for treatment clearly diverge and this effect is indeed significant, as shown below. In other words, fixed prostheses seem to pay off and outcompete partial prostheses in the long run. In both study groups, extractions require adaptations of the inserted prosthetic restorations. A group difference regarding the timing of the extractions was, therefore, not to be expected. For the following analysis, we refrain from interval censoring or other debatable corrective actions since we intended to capture the situations as they are. This is justified by the assumption that the study has been consistently performed and tests are assumed to be conservative with respect to standard clinical settings.

Approaching a duration of roughly 8 years, the gained advantage is again lost as the curves begin to converge, confirming the impression from the event plot Fig. 1a. The convergence towards the end of the 8-year time course may be due to a saturation phenomenon, i.e., complete loss of teeth. The ratio of the two stratified CSMFs along with their pointwise confidence intervals provide an objective quantification of the treatment effect as discussed in the following.

## Results

### Treatment effect based on cumulative mean ratio

**Fig. 4:**
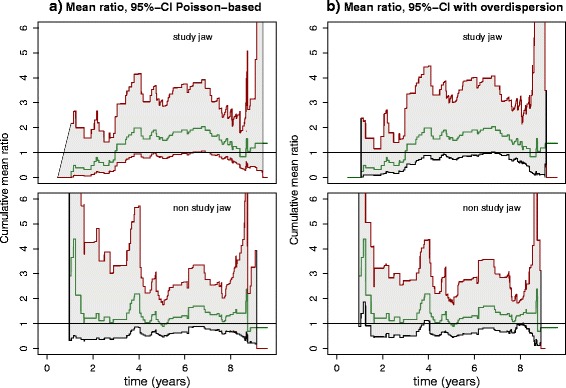
**a** Mean ratio functions with 95 % confidence regions with pointwise bounds based on a Poisson assumption. Top panel: SJ. Bottom panel: NSJ. **b** Mean ratio functions with 95 % confidence regions based on corrections for overdispersion. Top panel: SJ. Bottom panel: NSJ. *CI* confidence interval, *NSJ* non-study jaw, *SJ* study jaw

The ratio of the cumulative population mean functions, *μ*_1_(*t*) and *μ*_2_(*t*) for treatment 1 and 2, respectively,

5 $$ \psi(t) = \frac{\mu_{1}(t)}{\mu_{2}(t)}   $$

yields a measure of the treatment effect that can be easily estimated. Under a Poisson assumption for the event occurrences (Poisson process), the random variable

$$\log(\hat{\psi}(t)) = \log\left(\frac{\hat{\mu}_{1}(t)}{\hat{\mu}_{2}(t)}\right) $$

is approximately normally distributed, i.e.,

6 $$ N\left(\log(\hat\psi(t)),\frac{1}{r_{1}(t)\hat\mu_{1}}+\frac{1}{r_{2}(t)\hat\mu_{2}}\right),   $$

where *r*_1_(*t*) and *r*_2_(*t*) are the numbers of patients at risk at time *t* within the groups for treatment 1 and treatment 2, respectively (see Cook and Lawless [6], section 2.2.4). The time courses of $\hat {\psi }(t)$ for the SJ and the NSJ are shown in Fig. 4a along with the 95 % confidence regions estimated using Eqs. 5 and 6. The approximation given by Eq. 6 might not be justified. However, following [6] (section 2.2.4), $\hat \mu _{i}$, *i*=1,2, and $\log (\hat \mu _{i})$, *i*=1,2, are approximately normally distributed even if the counts are not Poisson random variables. This leads to the following more robust estimation. The relatively small difference between the results, however, justifies the use of Eq. 6 in retrospect.

Under the Poisson assumption, the variances should be roughly equal to the means, which is not fulfilled in the case at hand. The given estimates suggest overdispersion, so that the confidence region is more appropriately computed based on the assumption that $\log (\hat {\psi }(t))$ is approximately normally distributed with corrected variances, i.e.,

7 $$ N\left(\log(\hat\psi(t)),\frac{\hat\sigma_{1}}{r_{1}(t){\hat\mu_{1}^{2}}}+\frac{\hat\sigma_{2}}{r_{2}(t){\hat\mu_{2}^{2}}}\right).   $$

Thereby, $\hat \sigma _{i}$, *i*=1,2, are the sample variance estimates according to Eq. 4 for the two treatment groups (see [6], section 2.2.4). The time courses of $\hat {\psi }(t)$ taking overdispersion into account are shown in Fig. 4b along with the 95 % confidence regions estimated using Eqs. 5 and 7. It turns out, however, that overdispersion is rather weak in terms of the differing results from Eqs. 6 and 7, indicating that the deviation from a pure Poisson most likely stems from a time dependency of the recurrence rate addressed in the following section. It indeed turns out that the instantaneous recurrence rate is accelerated.

Under the presumption of equal treatment effects, the ratio of the cumulative mean functions should be equal to 1. It can be seen in the upper part of Fig. 4a that under the Poisson assumption, the confidence region for the SJ suggests there is a difference from the one-to-one ratio at the edge of statistical significance from roughly *t*=3.5 years on. This confirms the earlier impression gained from Fig. 3a. Taking the more accurate assumption of overdispersion into account, the effect is somewhat less pronounced but still clearly present. The advantage of treatment 2 (fixed prosthesis), even if only at the edge of statistical significance, is striking. The current scientific consensus states that treatment 1 is preferable to treatment 2. The replacement of lost molars is considered worthwhile, ignoring the well-known periodontal setbacks initiated by removable dental prostheses. Tooth loss is generally expected, but it is assumed that within a tightly controlled setting it can be minimized. That does not seem to be the case.

The effect of treatment seems to have a notable impact onto the NSJ as well, as can be seen in the lower panels of Fig. 4a and b. There is a tendency for the NSJs to follow the recurrence rates of the SJs. A possible interpretation is that an improved condition for the one jaw has a favorable effect on the remaining jaw. The NSJ cannot be used as a control group, i.e., to formulate a null hypothesis due to this effect.

### Three-phasic Poisson process

The Poisson density function for the probability of *y* recurrences is

8 $$ f(y) = \frac{1}{y!}(\lambda t)^{y} \exp(-\lambda t),   $$

where *λ* is the number of recurrences per time unit per population unit (tooth extractions per day per patient). Given a Poisson process, the CSMF is *M*(*t*)=*λt* (Nelson [5], chap. 8). The nonlinear curves in Fig. 3a immediately tell us that we have to reject this oversimplification. However, to keep the model as simple as possible, yet plausible, we introduce a three-phasic Poisson process, i.e., a piecewise time-independent (linear) process:

9 $$ M(t) = \left\{\begin{array}{ll} \lambda_{1} t, & t\leq t_{1}, \\ \lambda_{1} t_{1} + \lambda_{2} (t-t_{1}), & t_{1} < t \leq t_{2}, \\ \lambda_{1} t_{1} + \lambda_{2} (t_{2}-t_{1}) + \lambda_{3} (t-t_{2}), & t > t_{2}.\\ \end{array}\right.   $$

**Fig. 5:**
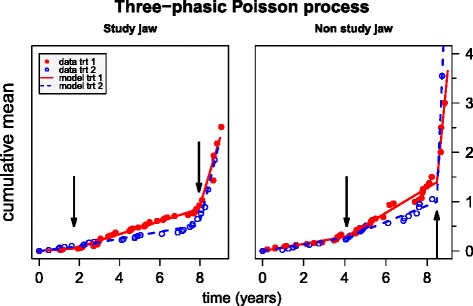
Three-phasic Poisson process (three-step piecewise linear regression). It is fitted to the cumulative mean functions along with an estimation of the two change points (indicated by *arrows*). The models are stratified for the treatment groups and fitted separately to the data of the SJ (left panel) and the NSJ (right panel). The upper left figure legend applies to both panels. *NSJ* non-study jaw, *SJ* study jaw, *trt* treatment

This piecewise linear function is fitted to the stratified CSMFs separately for the SJ and the NSJ using a maximum likelihood approach. Both the instantaneous recurrence rates, *λ*_1_, *λ*_2_, and *λ*_3_, as well as the interval boundaries, *t*_1_ and *t*_2_, are estimated using a Gaussian likelihood. The change points (*t*_1_,*t*_2_) for the two treatment strata are constrained to the same values to get pairwise comparable intervals. The estimated recurrence rates and the change points for the SJ as well as for the NSJ along with their confidence intervals are compiled in Table 1. The fitted piecewise linear functions are depicted in Fig. 5. The estimated change points are indicated by arrows. The bblme package in R has been used [9] for the estimation. This package also provides a rigorous estimation of confidence intervals based on a profile likelihood. The following significance evaluations are based on confidence in terms of checking overlaps of confidence intervals.

**Table 1:** Estimated recurrence rates (per year) and the change points (in years) for the study jaw and the non-study jaw, based on a tripartite Poisson process according to Eq. 9

	Estimate	2.5 %	97.5 %
Study jaw			
*λ*_1_, treatment 1	0.0284	−0.0586	0.06734
*λ*_1_, treatment 2	0.0497	0.0152	0.23034
*λ*_2_, treatment 1	0.1290	0.1167	0.1413
*λ*_2_, treatment 2	0.0709	0.0576	0.0855
*λ*_3_, treatment 1	1.3770	1.2665	1.5099
*λ*_3_, treatment 2	1.6490	1.4487	1.8816
*t*_1_	1.7350	−1.6202	2.7703
*t*_2_	7.9532	7.8942	8.0179
Non-study jaw			
*λ*_1_, treatment 1	0.0717	0.0589	0.0837
*λ*_1_, treatment 2	0.0665	0.0508	0.0788
*λ*_2_, treatment 1	0.2516	0.2324	0.2712
*λ*_2_, treatment 2	0.1654	0.1438	0.1874
*λ*_3_, treatment 1	4.2861	3.4070	5.1868
*λ*_3_, treatment 2	9.9602	7.6880	12.6717
*t*_1_	4.0901	3.5693	4.4598
*t*_2_	8.4751	8.3927	8.5269

### Discussion

The recurrence rate for the SJ in treatment group 1 starts with *λ*_1_=0.03 per year during the first time interval. After an estimated duration of *t*_1_=1.74 years, the recurrence rate increases more than fourfold to *λ*_2_=0.13 per year. At the estimated change point *t*_2_=7.95, the recurrence rate once more increases tenfold to *λ*_3_=1.38 per year.

The same trend holds for treatment group 2 but at a significantly lower recurrence rate of *λ*_2_=0.07 in the long middle interval between change points *t*_1_=1.74 and *t*_2_=7.95. In the beginning, until change point *t*_1_=1.74, the difference between the recurrence rates for the two treatment groups is insignificant. In other words, the two types of prosthesis seem to make no difference in protecting against loss of teeth during roughly the first 2 years. However, for the following 6 years, the protection is twice as good for prosthesis type 2.

From the estimated change point *t*_2_=7.95 years, the recurrence rates of the two treatment groups converge again and both rates are relatively high. During this last phase, the patients under treatment 2 lose slightly more teeth than the patients under treatment 1, on average. On the one hand, this result for the short third interval has to be taken with caution since the number of patients at risk at these late observation times is rather small. On the other hand, several patients lose a considerable number of teeth at once in the third interval. One patient lost 12 teeth at once at time *t*=8.73 years under treatment 2. It seems as if with treatment 2, tooth loss is delayed for some 7–8 years, but then catches up. The small number of patients who contribute to the last interval, due either to dropout from the study or to earlier complete loss of teeth, is reflected in the rather wide confidence intervals of the estimates for the recurrence rates. The increasing censoring towards the end of the study is also reflected in the increasing variance of the mean ratio function (see Fig. 4).

The largest instantaneous recurrence rate turns out to be *λ*_3_=9.96 per year for the third interval of the treatment 2 group of the NSJ. Note, however, that this estimation is based on a multiple concurrent extraction event of one patient only. The recurrence rates for the SJ are smaller than those for the NSJ, which suggests that both treatments are able to decrease the recurrence rates for tooth loss. However, to conceive of the NSJs as a control group is questionable since the conditions of the SJ impinge on the conditions of the NSJ, as can be seen from the aligned estimations of the recurrence rates. In other words, the conditions of the NSJ follow the conditions of the SJ, albeit less pronounced. Thus, the comparison of the SJ with the NSJ should be taken as qualitative evidence. Due to this lack of strict comparability, we refrained from constraining the estimations of the change points to the same values for both jaws.

The results using instantaneous recurrence rates under the assumption of a tripartite Poisson process confirm the insights gained from the confidence intervals of the mean ratios. Based on confidence, there is a significant difference between the effects of the two types of prosthesis in the intermediate period after insertion, i.e., treatment 2 is able to delay tooth loss for some 7–8 years. Specifically, the fixed prosthesis reduces the tooth loss rate by a factor of 1.82 from 0.13 per year to 0.07 per year compared to the partial removable prosthesis in the second phase.

## Conclusions

The approach based on cumulative history functions provides an appropriate method with an intuitively interpretable output for similar recurrent-event processes as given by the tooth extraction rate in this study. The main advantage of this method is that it is based on process, leading to an instantaneous intensity (recurrence rate) function. Based on a piecewise constant Poisson process assumption, a simple model function can be fitted to the observed time-dependent intensity function. The effects of predictors can be point- or interval-wise evaluated. For the case at hand, the recurrent-event process approximately underlies a tripartite Poisson process, whereby only the middle phase is determined by significantly different recurrence rates for the two treatment groups. Perhaps, the treatment effect is reversed in the long run, which can be detected in following up the patients for a further couple of years (the 10-year follow-up is under way). Under this assumption, the overall effect is nil, however, losing an essential insight that treatment 2 is able to delay tooth loss in the medium term.

Tooth loss appears to be an accelerated phenomenon, as can be seen particularly clearly in the right panel of Fig. 3a. The increase in tooth loss after roughly 3 years (visible through the step change in the CSMF) is somewhat decelerated by the prostheses (left panel of Fig. 3a). The strength of deceleration is different for the two treatment types.

We hope that this approach will contribute to an improved investigation of similar recurrent-event phenomena. The way we tackled the problem of a finite number of possible recurrences can perhaps be improved further. We hope that our work will stimulate further development of process-based approaches, since the approach of our study yielded relevant and interpretable results.

## Endnote

^1^ Patients initially recruited who never attended after recruitment have been removed from the analysis leaving 149 patients who took the survey.

## Additional file

Additional file 1 The flow chart of the clinical trial on which the article is based. (PPTX 69.7 kb)

## Abbreviations

CSMF: cumulative sample mean function
CSVF: cumulative sample variance function
NSJ: non-study jaw
SJ: study jaw
