# Supplementary material for: Process-based approach to modeling recurrent-event data explicated on the basis of occurrences of tooth losses in two different prosthetic treatment concepts
Source: Trials. 2016 May 17;17:244. doi: 10.1186/s13063-016-1360-y (PMC4869190; doi:10.1186/s13063-016-1360-y)
Supplement: Additional file 1 — The flow chart of the clinical trial on which the article is based. (PPTX 69.7 kb) [file 13063_2016_1360_MOESM1_ESM.pptx]

## Slide 1
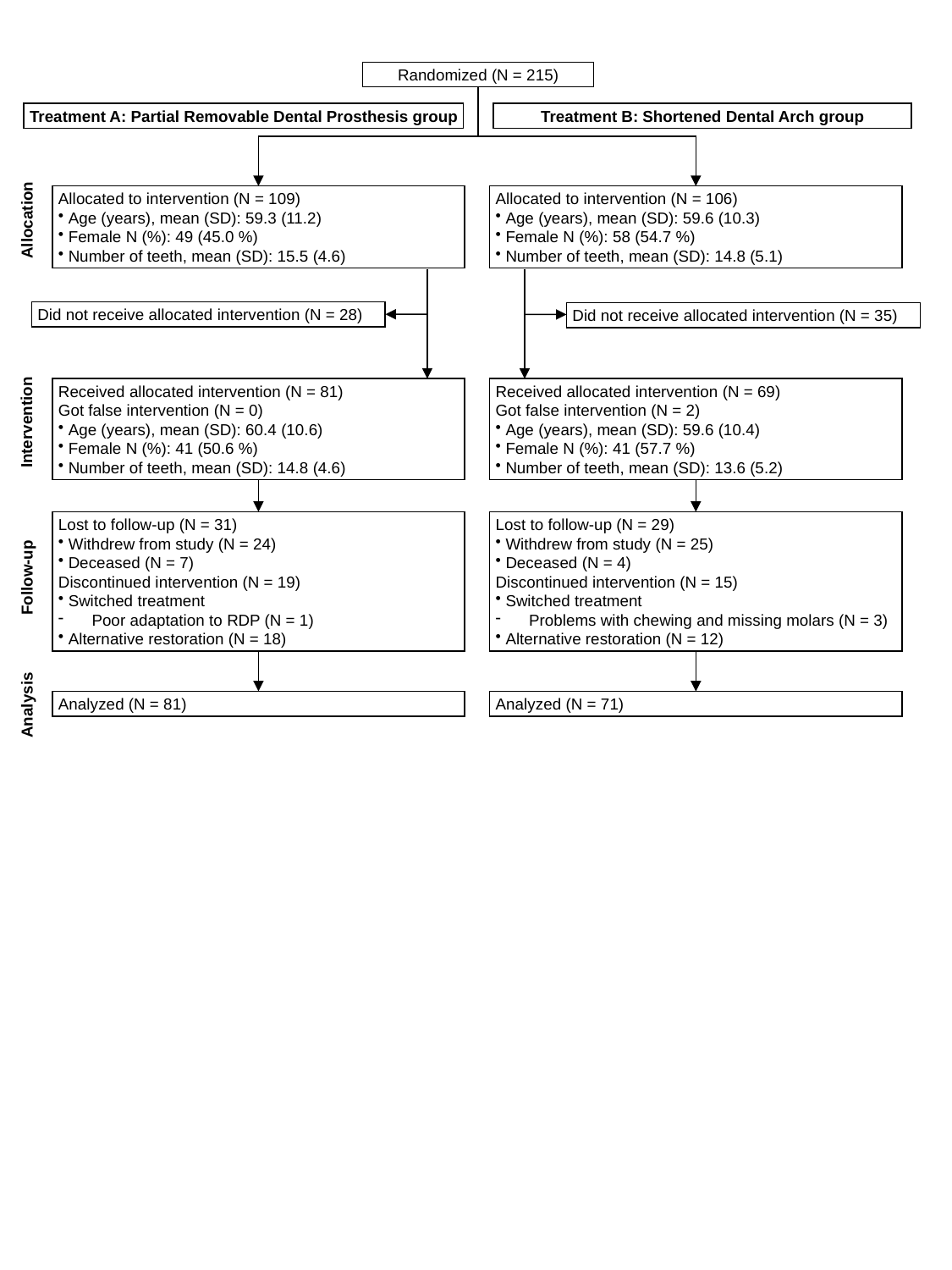

Randomized (N = 215)
Treatment A: Partial Removable Dental Prosthesis group
Treatment B: Shortened Dental Arch group
Allocated to intervention (N = 109)
 Age (years), mean (SD): 59.3 (11.2)
 Female N (%): 49 (45.0 %)
 Number of teeth, mean (SD): 15.5 (4.6)
Allocated to intervention (N = 106)
 Age (years), mean (SD): 59.6 (10.3)
 Female N (%): 58 (54.7 %)
 Number of teeth, mean (SD): 14.8 (5.1)
Allocation
Did not receive allocated intervention (N = 28)
Did not receive allocated intervention (N = 35)
Received allocated intervention (N = 81)
Got false intervention (N = 0)
 Age (years), mean (SD): 60.4 (10.6)
 Female N (%): 41 (50.6 %)
 Number of teeth, mean (SD): 14.8 (4.6)
Received allocated intervention (N = 69)
Got false intervention (N = 2)
 Age (years), mean (SD): 59.6 (10.4)
 Female N (%): 41 (57.7 %)
 Number of teeth, mean (SD): 13.6 (5.2)
Intervention
Lost to follow-up (N = 31)
 Withdrew from study (N = 24)
 Deceased (N = 7)
Discontinued intervention (N = 19)
 Switched treatment
 Poor adaptation to RDP (N = 1)
 Alternative restoration (N = 18)
Lost to follow-up (N = 29)
 Withdrew from study (N = 25)
 Deceased (N = 4)
Discontinued intervention (N = 15)
 Switched treatment
 Problems with chewing and missing molars (N = 3)
 Alternative restoration (N = 12)
Follow-up
Analyzed (N = 81)
Analyzed (N = 71)
Analysis
